# Metagenomic investigation of viruses in green sea turtles (*Chelonia mydas*)

**DOI:** 10.3389/fmicb.2025.1492038

**Published:** 2025-01-22

**Authors:** Hongwei Li, Yuan Chen, Zhongrong Xia, Daohua Zhuang, Feng Cong, Yue-Xiao Lian

**Affiliations:** ^1^School of Life Science, Huizhou University, Huizhou, China; ^2^Guangdong Huidong Sea Turtle National Nature Reserve Bureau, Sea Turtle Bay, Huizhou, China; ^3^State Key Laboratory for Conservation and Utilization of Bio-Resources in Yunnan, School of Life Sciences, Yunnan University, Kunming, China; ^4^Guangdong Laboratory Animal Monitoring Institute and Guangdong Provincial Key Laboratory of Laboratory Animals, Guangzhou, China

**Keywords:** green sea turtle, virome, metagenomics, feces, virus

## Abstract

Green sea turtles are listed on the International Union for Conservation of Nature’s Red List of Threatened Species. Thus, conservation efforts, including investigation of factors affecting the health of green sea turtles, are critical. Viral communities play vital roles in maintaining animal health. In the present study, shotgun metagenomics was used for the first time to survey viruses in the feces of green sea turtles. Most viral contigs were DNA viruses that mainly belonged to *Caudoviricetes*, followed by *Crassvirales*. Additionally, most of the viral contigs were not assigned to any known family or genus, implying a large knowledge gap in the taxonomy of green sea turtle gut viruses. Host prediction showed that most viruses were connected to two phyla: *Bacteroidetes* and *Firmicutes*. Furthermore, KEGG enrichment analysis showed that the viral genes were mainly involved in phage-associated and metabolic pathways. Phylogenetic tree reconstruction of *Caudovirales* terminase large-subunit (TerL) protein showed that most of the sequences were phylogenetically distant. This study expands our understanding of the viral diversity in green sea turtles. In particular, analysis of the virome RNA fraction is exceedingly important for investigating intestinal viromes; therefore, future studies could use metatranscriptomics to study RNA viruses.

## Introduction

Green sea turtles (*Chelonia mydas*) are the only herbivorous sea turtles that feed on seagrass and algae (Bjorndal, 1985). Green sea turtles are classified as “Vulnerable” owing to the intense commercial exploitation of their shell and meat and degradation of their nesting and marine habitats. They are listed on the International Union for Conservation of Nature’s Red List of Threatened Species under various statuses (International Union for Conservation of Nature, 2013). Consequently, there is an urgent need to establish nature reserves in which green sea turtles can be cared for, rehabilitated, or bred from wild individuals. The Huidong Sea Turtle National Nature Reserve was established in China for this purpose. However, changes in the habitat of these turtles may increase their risk of infectious diseases and hinder conservation efforts. Therefore, the future of green sea turtles partly depends on the development of protective measures against infectious diseases, particularly viral infections.

Viruses in the microbiota are highly diverse and infect both eukaryotic and prokaryotic cells as obligate parasites. Studies have shown that viral populations are not subject to periodic fluctuations (Minot et al., 2013; Shkoporov et al., 2019) but show marked inter-individual variation (Shkoporov et al., 2019). However, in healthy Westerners, the gut virome exhibits an age-dependent pattern (Gregory et al., 2020). Moreover, viral composition can be partially affected by dietary habits and the surrounding environment (Li et al., 2021). An increasing number of studies have indicated that the enteric virome plays a vital role in homeostatic regulation and disease progression through virus–microbiome and virus–host interactions (Virgin, 2014; Pfeiffer and Virgin, 2016; Mirzaei and Maurice, 2017; Mukhopadhya et al., 2019). However, multiple studies of the bacterial content of green sea turtle feces have been reported (Ahasan et al., 2017; Price et al., 2017; Ahasan et al., 2018; Campos et al., 2018; McDermid et al., 2020; Scheelings et al., 2020), no data on their virome are available. Therefore, investigating the viral communities in green sea turtles and identifying putative pathogens that threaten their health is imperative.

Unlike bacteria, viruses lack universal markers, making it difficult to identify unknown viruses. Recent advances in the sequencing and analysis of metaviromes have helped overcome this difficulty, adding to our understanding of the richness and complexity of the enteric virome (Garmaeva et al., 2019; Santiago-Rodriguez and Hollister, 2019). Additionally, metagenomics has been widely used to investigate the diversity of domestic animal fecal viromes, helping to elucidate the etiology of diarrheal diseases in livestock and identify potential zoonotic and emerging viruses (Mihalov-Kovács et al., 2014). In this study, we aimed to use metagenomic approaches to survey the enteric virome in healthy green sea turtles.

## Materials and methods

### Animals and sampling

Sample collection and all experiments were conducted in accordance with the guidelines established by the Committee on Biomedical Ethics of Huizhou University. All samples were collected in July 2021 from healthy green sea turtles, including three juvenile (approximately 7 years old, named GT44, GT66, and GT77), six subadult sea turtles (approximately 15 years old, named GT111, GT222, GT333, GT88, GT99, and GT1010), and one adult individual (approximately 30 years old, named GT55), all of which resided in the Sea Turtle National Nature Reserve, Huidong, China (22°33.15′N; 114°52.33′E). The turtles were reared from hatched eggs of wild females, which were reproduced by laying eggs on land in the reserve from July to October every year. The turtles were fed in the reserve and kept in tanks separated by a septum. All turtles shared circulating seawater. Green sea turtles were fed plant-based food containing a small amount of fish (10% fish, 90% plant-based). Fecal samples were collected from each turtle near their feeding time (stools produced after feeding). The core of each fecal pellet was collected using sterile forceps. The fecal samples were immediately placed into cryogenic vials and stored at −80°C until used in further experiments.

### Virus enrichment, purification, and viral metagenomics sequencing

The fecal samples were thawed and resuspended in 10 volumes of phosphate-buffered saline. The contents were vigorously vortexed for 5 min and then centrifuged at 15,000 × g for 10 min at 4°C to precipitate the particulate material. The collected supernatant was filtered through a 0.45-μm filter (Millipore) to remove eukaryotic and bacterial cell-sized particles. Following a previous report (Zhang et al., 2014), filtrate-enriched viral particles were treated with DNase and RNase to digest the unprotected nucleic acids at 37°C for 60 min. Total nucleic acids were extracted from the filtered supernatants using a TGuide S32 automatic nucleic acid extractor (Tiangen, China). Total nucleic acid extracts were reverse transcribed using SuperScript III (Thermo Fisher Scientific, Waltham, MA, United States) with random hexamers. cDNA was treated with RNase H before second-strand synthesis using a Klenow fragment (New England Biolabs, Ipswich, MA, United States). Treated DNA was quantified (Nanodrop) and randomly fragmented by ultrasonication (Covaris), followed by library construction. The DNA fragments were then endpolished, A-tailed, and ligated with the full-length adapter before being amplified further using polymerase chain reaction (PCR) amplification. PCR products were purified using AMPure XPsystem (Beckman Coulter, Beverly, United States). Subsequently, library quality was assessed on the Agilent 5400 system (AATI) and quantified using real-time PCR (1.5 nM). The qualified libraries were pooled and sequenced on Illumina platforms with PE150 strategy in Novogene Bioinformatics Technology Co., Ltd. (Novogene, Beijing, China).

### Read pre-processing

The original fluorescence image files obtained from the Illumina platform were transformed into short reads (raw data) by base calling and further converted into the FASTQ format using PICARD.^1^ Low-quality and short reads (with a minimum Phred score of 20 across the entire read length used as a quality cutoff), reads containing more than 10% N, overrepresented sequences, and adapters were removed using the Trimmomatic (v.0.39) tool (Bolger et al., 2014). Reads shorter than 75 bp were removed by trimming. To eliminate the host sequence, reads aligned to the reference *Chelonia mydas* genome (GCA_030012505.1) using the Bowtie2 (v.2.2.5) tool were excluded from subsequent analyses (Langmead and Salzberg, 2012). After the above read pre-processing, high-quality host-free reads remained. Trimmomatic and Bowtie2 were used with their default parameter settings.

### Bioinformatics analyses

The remaining high-quality reads were subsequently assembled using the metaSPAdes (v3.13.0) module in metawrap (v1.3.2, -m 300 -t 36 -l 200), which were selected because of their good performance in a comparative study of metagenomic assemblers (Nurk et al., 2017). VirSorter2 (v2.2.3) with the parameter settings “–include-groups dsDNAphage, NCLDV, RNA, ssDNA, lavidaviridae –min-length 1,000 –min-score 0.5 –seqname-suffix-off –viral-gene-enrich-off –provirus-off –prep-for-dramv” (Guo et al., 2021) in combination with the default parameter settings in VirFinder (v1.1) (Ren et al., 2017) was used to identify the assembled reads. Contigs were considered a candidate viral sequence: with at least one identified viral gene, a VirSorter2 score > 0.5, or a VirFinder score > 0.7. Candidate viral contigs were passed through the CheckV (v1.0.1) end_to_end model (Nayfach et al., 2021a,b) to assess genome completeness. All subsequent analyses focused on genomes with >50% completeness to avoid the limitations associated with small genome fragments. Viral contigs larger than or equal to 10 kb in length were selected for de-replication using the dRep (v3.4.5) tool (Olm et al., 2017) with the parameter settings (–length 10,000 –P_ani 0.90 –S_ani 0.95 –cov_thresh 0.3). Then, representative viral contigs with 95% identity were used for subsequent analyses. Taxonomic assignment of the viral genomes was performed using geNomad (1.7.6) (Camargo et al., 2023). The abundance of virus sequences was determined based on the average depth of the contigs using Bowtie2. iPHoP (v1.3.3),^2^ a tool for integrated phage-host prediction, was used to maximize host prediction for metagenome-derived viruses of archaea and bacteria, with a confidence score > 90% at the genus level (Roux et al., 2023). Prodigal (v2.6.3),^3^ a gene-finding program for microbial genomes, was used to predict genes based on viral contigs (Hyatt et al., 2010). The phage terminase large-subunit domain (TerL) was utilized to construct phylogenetic trees using BITACORA (v1.4) (Vizueta et al., 2020). Here, geNomad, Bowtie2, Prodigal, and BITACORA all use the default parameter settings. MAFFT (v6.240) was used to align sequences with default parameter settings, trimAl (v1.5) was used to remove low-quality aligned sequences, and FastTree (v2.1.11) was used to construct the maximum likelihood tree. The bootstrap values for each branch are included in the Supplementary material (TerL.unique.gt100aa.gt50.bt1000.treefile). Taxonomic annotation below the phage order level follows the MGV algorithm (Nayfach et al., 2021a,b). Clustering was performed using mcl (v14.137), and phage sequences obtained in this study were clustered at the family and genus levels with phage proteins from the INfrastructure for a PHAge REference Database (INPHARED, v3Dec2023). For each cluster, the family or genus was assigned based on the most frequently represented family or genus among known phages within the cluster. Phages without a defined family or genus in the database were labeled as “Unclassified,” while those that could not be assigned to any cluster were labeled as “Unclustered.”

Principal coordinate analysis (PCoA) was performed using Canberra distance matrices. KEGG pathway analyses were performed using the Databases for Annotation, Visualization, and Integrated Discovery (DAVID version 6.8) and KEGG (KOBAS version 3.0). The Mann–Whitney *U* test was used to determine the significance of differences between groups. Statistical significance was set at *p* < 0.05. A contingency table was constructed using the number of KEGG Orthologies (KOs) predicted to belong to or not belong to each pathway based on the viral genomes, alongside the number of KOs belonging to or not belonging to each pathway in the entire KEGG database. Enrichment analysis was then conducted using Fisher’s exact test.

## Results

### Genomic catalog of DNA viruses from the green sea turtle gut microbiome

**Figure 1:**
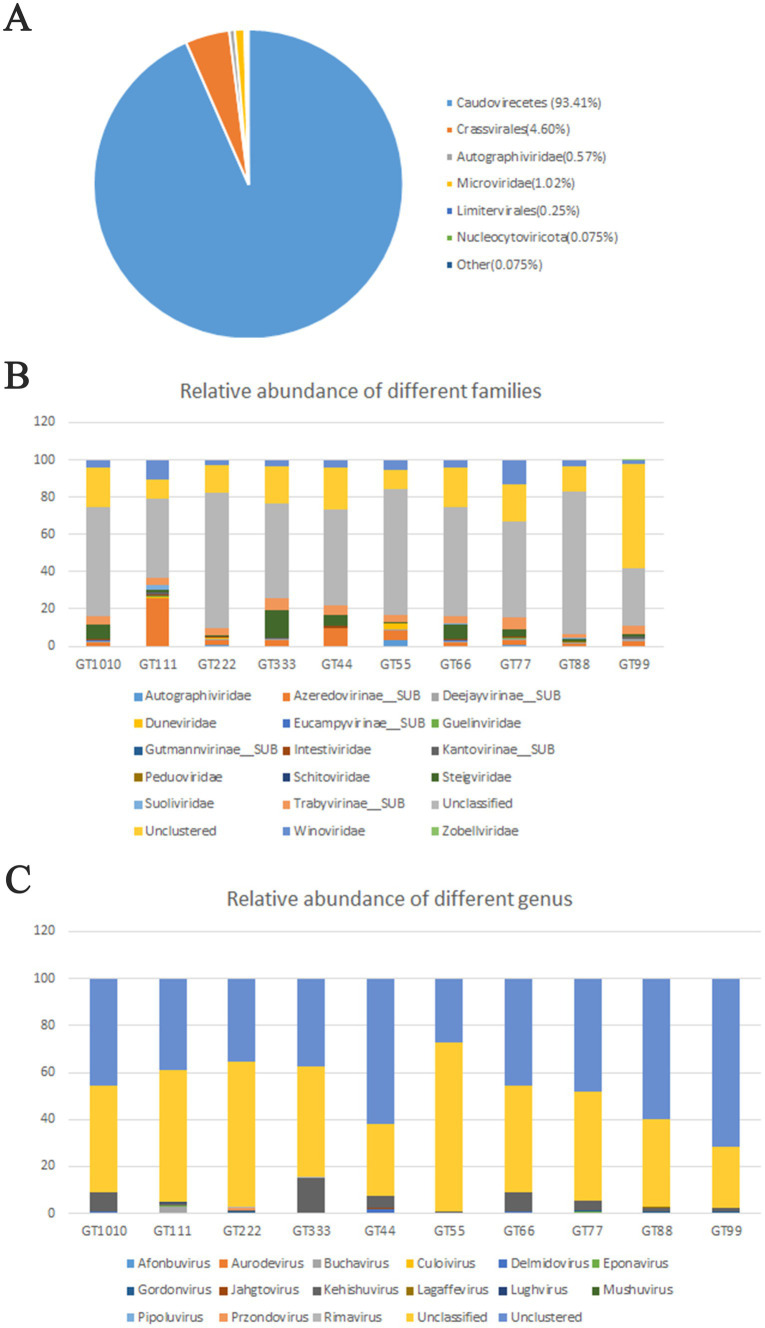
Taxonomic assignment of metagenomic reads. **(A)** 1,564 contigs were annotated taxonomically, most of which are DNA viruses, belonging to *Caudoviricetes* (93.41%), followed by *Crassvirales* (4.60%), *Microviridae* (1.02%), *Autographiviridae* (0.57%), and *Limitervirales* (0.25%). **(B)** Fecal virome composition was investigated at a family level in all green sea turtles. The top two families were *Unclassified* and *Unclustered*, followed by *Azeredovirinae*, *Winoviridae*, *Steigviridae*, and *Trabyvirinae* (the average relative abundances were 56.12, 20.87, 5.80, 5.42, 4.53, and 4.34%, respectively). **(C)** At the genus level, the top two families were *Unclassified* and *Unclustered*, followed by *Azeredovirinae*, and the others (the average relative abundances were 46.70, 47.24, 4.50%, and less than 0.3%, respectively).

**Figure 2:**
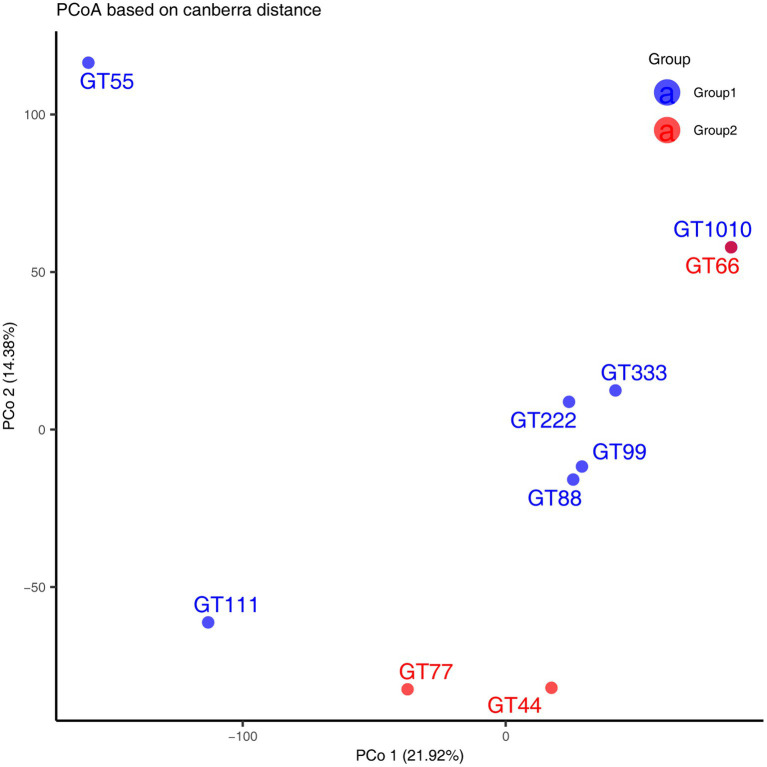
Principal Coordinate Analysis (PCoA) was performed using Canberra distance matrices. GT1010, GT111, GT222, GT333, GT44, GT55, GT66, GT77, GT88, and GT99 represent different green sea turtle samples. Group 1 consists of GT1010, GT111, GT222, GT333, GT55, GT88, and GT99; Group 2 consists of GT44, GT66, and GT77.

A total of 1069.4 million high-quality reads remained in the 10 virome data collections after quality filtering (mean = 106.94 million/sample, ranging from 101.07 to 121.2 million), which were assembled *de novo* (Supplementary Excel S1). After assembly, 937,157 contigs were generated and the average length of contigs was 3,178 bp (Supplementary Excel S2). After viral identification through VirSorter2 in combination with VirFinder2 and assessment of viral genomes’ completeness by CheckV (with >50% completeness to avoid limitations associated with small genome fragments), 1,564 contigs were annotated taxonomically, most of which were DNA viruses, belonging to *Caudoviricetes* (93.41%), followed by *Crassvirales* (4.60%), *Microviridae* (1.02%), *Autographiviridae* (0.57%), and *Limitervirales* (0.25%) (Figure 1A); however, in GT111, one contig was classified as *Nucleocytoviricota*. Nucleocytoviricota viruses belong to a newly established phylum, originally grouped as nucleocytoplasmic large DNA viruses. To perform fast and accurate genomic comparisons, the viral contigs larger or equal to 10 kb were selected to de-replicate. After de-replication, 751 representative viral genomes were acquired (Supplementary Excel S3) for subsequent analysis. Based on representative viral genomes, the fecal virome composition was investigated at the family and genus levels in all samples. At the family level, in all green sea turtles, the top two families were *the Unclassified* (average relative abundance, 56.12%) and *the Unclustered* (average relative abundance, 20.87%), followed by *Azeredovirinae* (average relative abundance, 5.80%), *Winoviridae* (average relative abundance, 5.42%), *Steigviridae* (average relative abundance, 4.53%), and *Trabyvirinae* (average relative abundance, 4.34%) (Figure 1B). At the genus level, the top two genera were *the Unclassified* (average relative abundance, 46.70%) and *the Unclustered* (average relative abundance, 47.24%), followed by *Azeredovirinae* (average relative abundance, 4.50%) and others (average relative abundance less than 0.3%) (Figure 1C). The results revealed that most viral contigs were not assigned to any known family or genus, confirming a large knowledge gap in the taxonomy of green sea turtle gut viruses. Additionally, the PCoA results did not show marked segregation between groups but showed obvious segregation among individuals (Figure 2).

### Host prediction, KEGG enrichment, and evolutionary analysis

**Figure 3:**
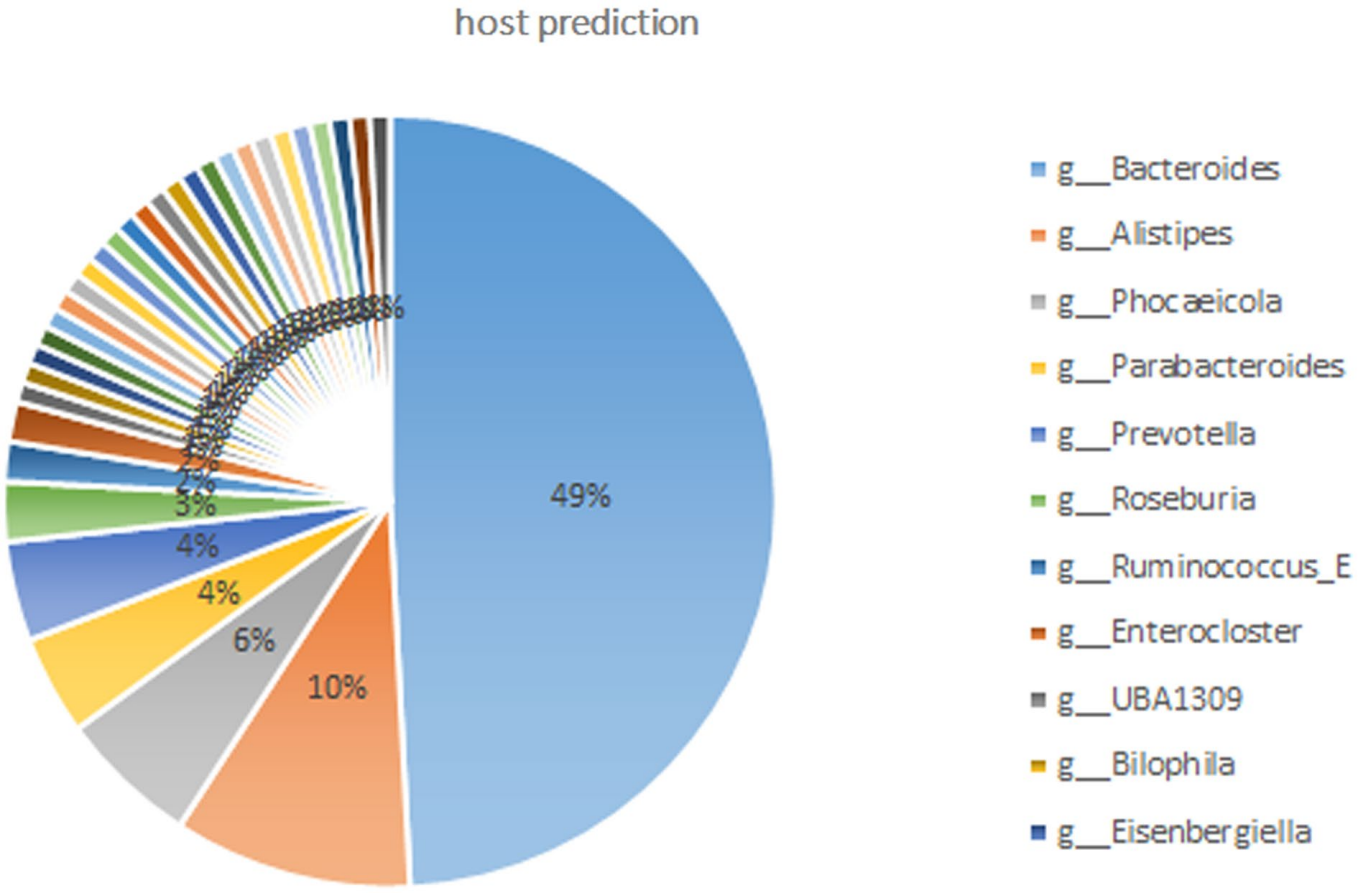
Host predictions at the genus level through iPHoP (An integrated machine learning Framework). Only the high-confidence host genus prediction (estimated <10% false discovery rate (FDR) or iPHoP score ≥ 90) was considered. Viruses were mainly connected to the phylum *Bacteroidia* and included the genera *Bacteroides*, *Alistipes*, *Phocaeicola*, *Parabacteroides*, and *Prevotella*; other genera belonged to the phylum *Firmicutes*.

**Figure 4:**
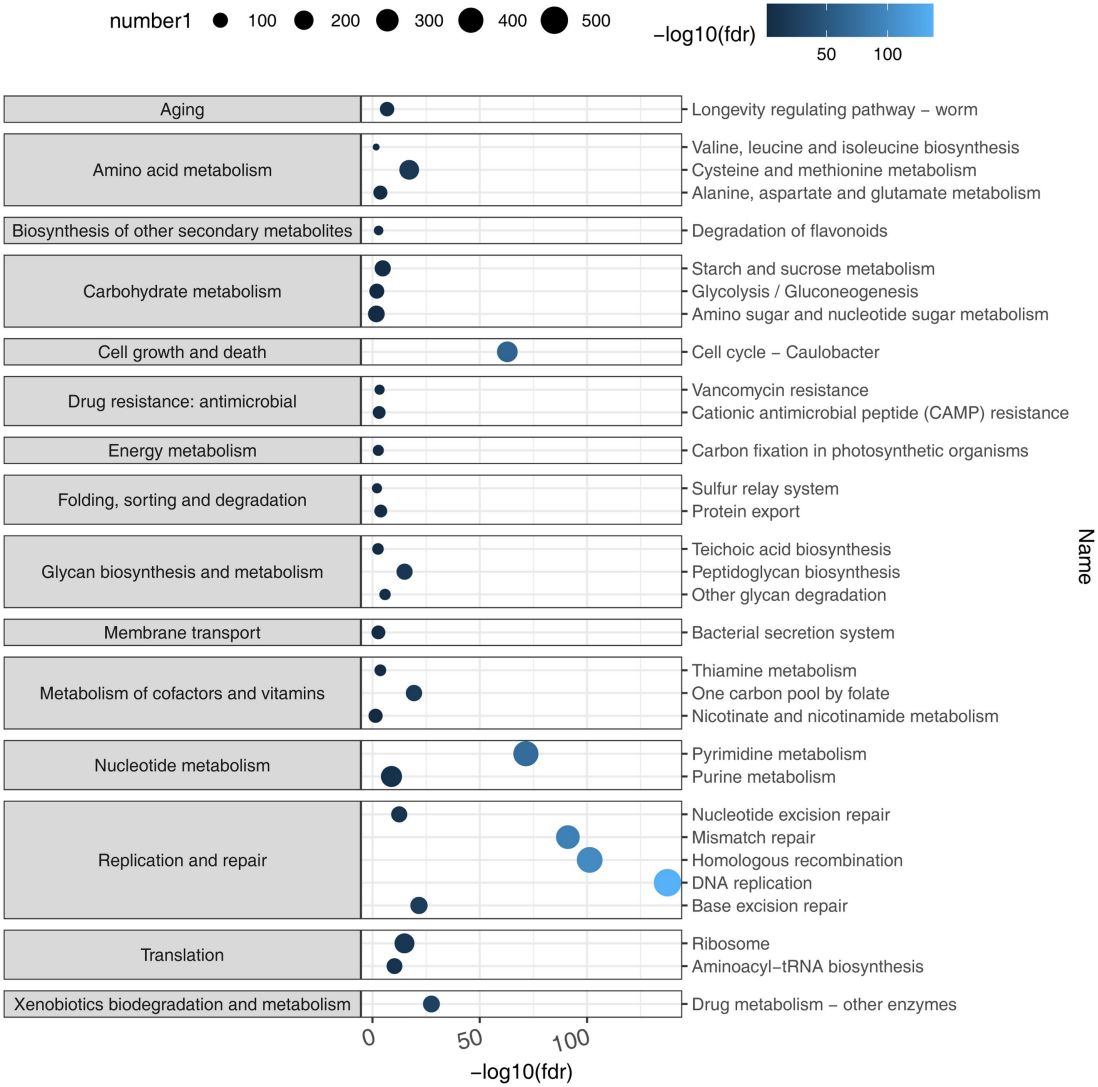
KEGG enrichment analysis. By taking the negative logarithm of fdr (with a base of 10), it can be converted to a positive number and the differences between smaller fdr values can be amplified, making the results easier to interpret and compare using the number of KEGG Orthologies (KOs) predicted to belong to or not belong to each pathway based on the viral genomes. Viral genes were mainly involved in the pathways that included DNA replication, homologous recombination, pyrimidine metabolism, mismatch repair, purine metabolism, cell cycle, ribosome, and cysteine and methionine metabolism.

Based on the representative viral genomes, iPHoP was used to yield highly accurate predictions at the genus level, with only high-confidence host genus prediction (estimated < 10% FDR or iPHoP score ≥ 90) remaining (Supplementary Excel S4). A total of 121 viruses were mainly associated with *Bacteroides*, *Alistipes*, *Phocaeicola*, *Parabacteroides*, and *Prevotella* (Figure 3), which belong to the phylum *Bacteroidia* and other viruses mainly belonging to the phylum *Firmicutes*. These two phyla were the dominant bacteria in the gut microbiome. These results show that host–virus interactions can be systematically elucidated through the extensive assembly of both viral and microbial genomes from the same environment. Furthermore, KEGG enrichment analysis showed that viral genes were mainly involved in the pathways of DNA replication, homologous recombination, pyrimidine metabolism, mismatch repair, purine metabolism, cell cycle, ribosomes, and cysteine and methionine metabolism (Figure 4), which revealed a relatively high frequency of phage-associated and metabolic genes.

**Figure 5:**
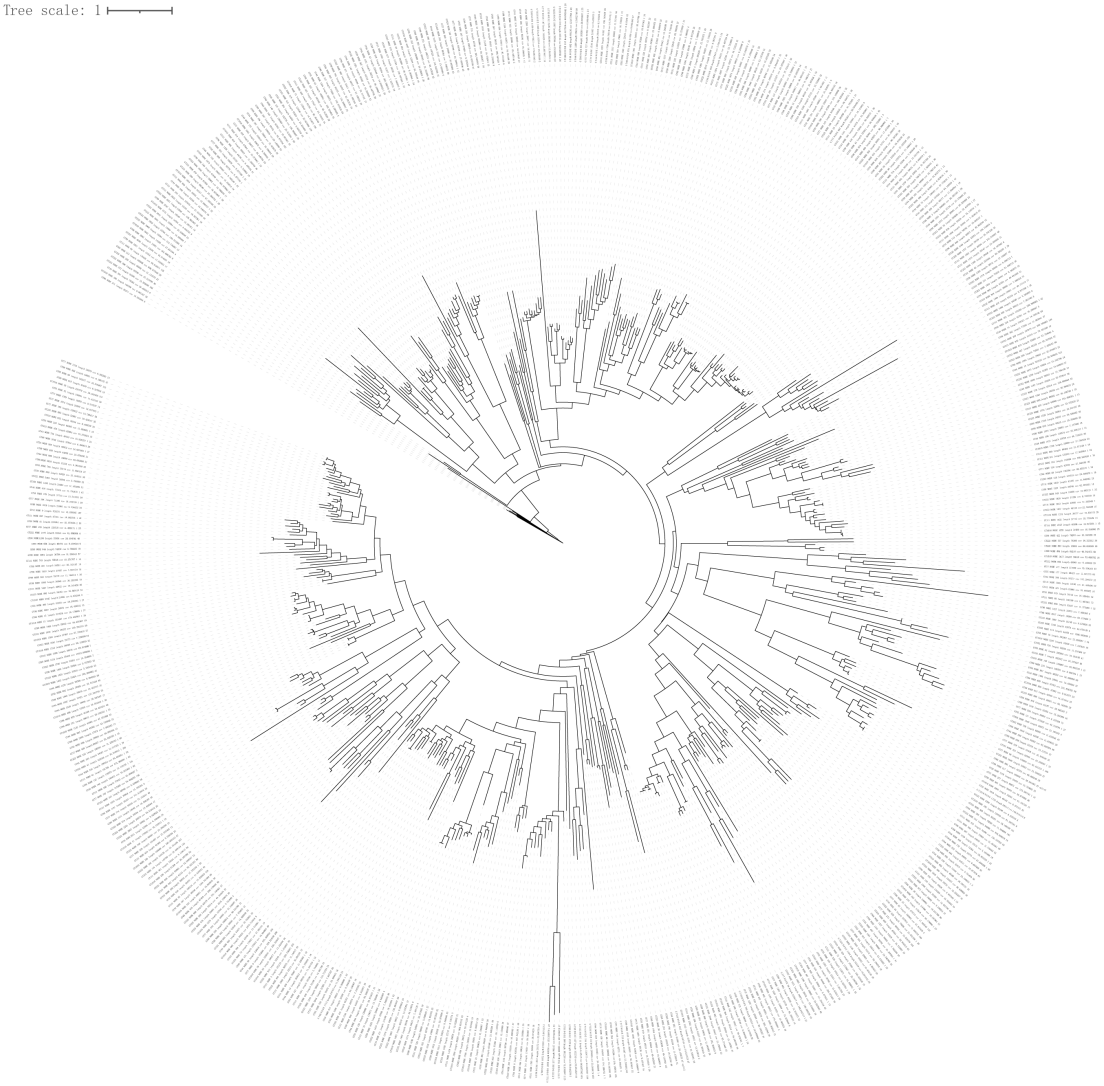
Phylogenetic tree analysis. To infer virus phylogeny, a total of 576 large terminase (TerL) protein sequences (containing >100 amino acids; only the largest TerL protein being selected for constructing phylogenetic tree when many TerL proteins were present in the representative viral genomes) were used to construct phylogenetic tree using the BITACORA tool.

To infer virus phylogeny, a total of 576 large terminase (TerL) protein sequences (containing >100 amino acids, only the largest TerL protein was selected for constructing a phylogenetic tree when many TerL proteins were contained in the representative viral genomes) were used to construct a phylogenetic tree using the BITACORA tool. The phylogenetic tree based on phage TerL amino acid sequences is shown in Figure 5. This tree reveals the evolutionary relationships of TerL sequences from different viral genomes, highlighting their broad distribution and indicating the viral diversity within the green sea turtle gut virome.

## Discussion

Recently, viral metagenomics has been widely used to identify animal viruses (Shan et al., 2011; Zhang et al., 2014; Li et al., 2015; Scheel et al., 2015; Kluge et al., 2016; Wu et al., 2016; Zhang et al., 2017; D’arc et al., 2018). Viral metagenomics can provide information regarding the composition of animal viromes, investigate the etiology of infectious diseases, and identify zoonotic and emerging viruses (Mihalov-Kovács et al., 2014; Zhang et al., 2017). In the present study, we analyzed, for the first time, the gut viromes of 10 green sea turtles of different ages.

The most abundant viruses in all samples were bacteriophages (viruses that infect bacteria), which constitute the majority of viral particles (Nayfach et al., 2021a,b) that affect microbial ecosystem processes through phage predation, lysogeny, and horizontal gene transfer. In this study, most bacteriophages were *Caudoviricetes*, followed by *Crassvirales*. Consistent with this, *Caudovirales,* belonging to the class *Caudoviricetes*, are highly represented in stool metagenomes (Nayfach et al., 2021a,b). *Crassvirales* (crAss-like phages) also belong to the class *Caudoviricetes* and are an abundant group of human gut-specific bacteriophages (Ramos-Barbero et al., 2023). In this study, most viral contigs were not assigned to any known family or genus, indicating that viral taxonomy is not as well defined as prokaryotic taxonomy. This may be explained by the fact that many taxonomic assignments are based on genetic distance-based clustering methods that depend on the chosen identity cutoffs (Pinto et al., 2024). Green turtles are herbivorous sea turtles, and the dominant phyla in their gut microbiota are *Firmicutes* and *Bacteroidetes* (Campos et al., 2018; McDermid et al., 2020). In this study, host prediction showed that most viruses were connected to the two phyla, suggesting that host–virus linkages may be important for understanding disease processes and host–virus co-evolutionary dynamics. In this study, only 33% of the viral genes were assigned preliminary biological functions. These genes are mainly involved in phage-associated and metabolic pathways. In the future, elaborative efforts and novel procedures are needed to predict protein function in viral genomes using deep learning (Kulmanov and Hoehndorf, 2020) and functional metagenomic assays (Schmitz et al., 2010). The *TerL* gene, as a conserved gene of bacteriophage viruses, is widely used in the construction of bacteriophage evolutionary trees (Nayfach et al., 2021a,b; Kavagutti et al., 2023; de Jonge et al., 2024). *TerL* is highly conserved in *Caudoviruses* and has often been used to infer their phylogeny (Desiere et al., 2001; Casjens et al., 2005; Mizuno et al., 2013). Phylogenetic tree reconstruction of *Caudoviruses* TerL protein showed that most sequences were phylogenetically distant, suggesting an uncharacterized diversity of the gut virome in green sea turtles.

In this study, the viral composition and the relative abundance did not display notable differences among different age groups but showed marked inter-individual variation. Similarly, a study reported that human viral populations are not subject to periodic fluctuations but instead show large inter-individual diversity (Shkoporov et al., 2019). Substantial inter-individual variations in the virome appear to be largely caused by environmental factors rather than genetic factors (Garmaeva et al., 2019). For example, co-twins (monozygotic twins) do not share more virotypes than unrelated individuals (Moreno-Gallego et al., 2019). However, our ability to explore the effects of age on viral diversity in green sea turtles was restricted because we could not compare the viral communities of adult turtles with those of subadult and juvenile turtles; only one adult turtle was sampled. Further investigation is needed to expand the adult green sea turtle samples, which can be used to compare viral communities among older age groups.

We speculate that the viral composition of green turtles could be partially affected by diet, as the turtles in our study were fed a plant-based diet through which insect or plant viruses could have been transmitted. Similarly, in humans, the composition of the human gut virome was firmly associated with diet, and individuals on the same diet showed a more similar virome composition (Minot et al., 2011). Additionally, the viral communities of green turtles could be affected by aquarium environments. In summary, diet potentially mediates the virome by driving microbiome fluctuations (Oh et al., 2019; Sausset et al., 2020). In this study, the turtles shared both diet and seawater in the tanks; thus, it is highly likely that they shared the same microbial communities in terms of viruses. Further studies are warranted to assess the source of viruses (seawater or food) by exploring viral communities in seawater and in their diet. Furthermore, we speculate that frequent contact with feeders might introduce human cutaneous viruses into the green sea turtles, suggesting that feeders should wear gloves when feeding turtles to avoid introducing cutaneous viruses.

In this study, shotgun metagenomics was performed to explore the green sea turtle fecal virome. This method is an optimal tool for studying the virome of DNA viruses (Gregory et al., 2022; Pandolfo et al., 2022; Shen et al., 2023) but is not insufficient for RNA viruses. In particular, analysis of the virome RNA fraction is exceedingly important for the unraveling of intestinal viromes. Therefore, future studies could use metatranscriptomics to study RNA viruses.

Finally, our study has some limitations. The small number of individuals investigated may have produced imprecise estimates of virome profiles. However, this study provides preliminary information for further exploration of putative pathogens in green sea turtles. A larger number of samples will be necessary to reduce the intrinsic sampling variation in the future. Moreover, our samples did not allow us to identify the virome of wild green sea turtles because all samples were collected from green sea turtles in a Nature Reserve. Further studies are needed to comparatively analyze the fecal viromes of captive and wild green sea turtles to identify and differentiate between the original and introduced viruses.

## Conclusion

This study describes, for the first time, the fecal virome of green sea turtles, although these preliminary observations were conducted in a small group. Our results indicate that the fecal viromes of green sea turtles are mainly composed of DNA viruses, among which *Caudoviricetes* dominate or co-dominates among the samples. Host prediction showed that most viruses belonged to two phyla, *Bacteroidetes* and *Firmicutes*. Furthermore, KEGG enrichment analysis showed that the viral genes were mainly involved in phage-associated and metabolic pathways. Phylogenetic tree reconstruction of the caudovirus TerL protein showed that most sequences were phylogenetically distant, suggesting an uncharacterized diversity of the gut virome in green sea turtles.

## Data Availability

The datasets presented in this study can be found in online repositories. The names of the repository/repositories and accession number(s) can be found in the article/Supplementary material.

## Footnotes

^1^ http://picard.sourceforge.net

^2^Available at https://bitbucket.org/srouxjgi/iphop.

^3^ http://compbio.ornl.gov/Prodigal/
